# System-Level Factors Associated With Telephone and Video Visit Use: Survey of Safety-Net Clinicians During the Early Phase of the COVID-19 Pandemic

**DOI:** 10.2196/34088

**Published:** 2022-03-10

**Authors:** Anjana E Sharma, Elaine C Khoong, Maribel Sierra, Natalie A Rivadeneira, Malini A Nijagal, George Su, Courtney R Lyles, Triveni DeFries, Delphine S Tuot, Urmimala Sarkar

**Affiliations:** 1 Department of Family & Community Medicine University of California San Francisco San Francisco, CA United States; 2 Center for Vulnerable Populations Zuckerberg San Francisco General Hospital University of California San Francisco San Francisco, CA United States; 3 Division of General Internal Medicine, Zuckerberg San Francisco General Hospital Department of Medicine University of California San Francisco San Francisco, CA United States; 4 Department of Obstetrics and Gynecology University of California San Francisco San Francisco, CA United States; 5 Division of Pulmonary, Critical Care, and Sleep, Zuckerberg San Francisco General Hospital Department of Medicine University of California San Francisco San Francisco, CA United States; 6 Department of Nephrology University of California San Francisco San Francisco, CA United States

**Keywords:** telemedicine, safety-net hospitals, health care delivery, ambulatory care, vulnerable populations, COVID-19, survey, vulnerable, telehealth, hospital, safety, delivery, video, implementation, health system

## Abstract

**Background:**

The COVID-19 pandemic prompted safety-net health care systems to rapidly implement telemedicine services with little prior experience, causing disparities in access to virtual visits. While much attention has been given to patient barriers, less is known regarding system-level factors influencing telephone versus video-visit adoption. As telemedicine remains a preferred service for patients and providers, and reimbursement parity will not continue for audio visits, health systems must evaluate how to support higher-quality video visit access.

**Objective:**

This study aimed to assess health system–level factors and their impact on telephone and video visit adoption to inform sustainability of telemedicine for ambulatory safety-net sites.

**Methods:**

We conducted a cross-sectional survey among ambulatory care clinicians at a hospital-linked ambulatory clinic network serving a diverse, publicly insured patient population between May 28 and July 14, 2020. We conducted bivariate analyses assessing health care system–level factors associated with (1) high telephone adoption (4 or more visits on average per session); and (2) video visit adoption (at least 1 video visit on average per session).

**Results:**

We collected 311 responses from 643 eligible clinicians, yielding a response rate of 48.4%. Clinician respondents (N=311) included 34.7% (n=108) primary or urgent care, 35.1% (n=109) medical, and 7.4% (n=23) surgical specialties. Our sample included 178 (57.2%) high telephone adopters and 81 (26.05%) video adopters. Among high telephone adopters, 72.2% utilized personal devices for telemedicine (vs 59.0% of low telephone adopters, *P*=.04). Video nonadopters requested more training in technical aspects than adopters (49.6% vs 27.2%, *P*<.001). Primary or urgent care had the highest proportion of high telephone adoption (84.3%, compared to 50.4% of medical and 37.5% of surgical specialties, *P*<.001). Medical specialties had the highest proportion of video adoption (39.1%, compared to 14.8% of primary care and 12.5% of surgical specialties, *P*<.001).

**Conclusions:**

Personal device access and department specialty were major factors associated with high telephone and video visit adoption among safety-net clinicians. Desire for training was associated with lower video visit use. Secure device access, clinician technical trainings, and department-wide assessments are priorities for safety-net systems implementing telemedicine.

## Introduction

The COVID-19 pandemic catalyzed a dramatic increase in telemedicine care, prompted by the need for physical distancing and Medicare and Medicaid reimbursement changes enabling parity in coverage for telemedicine [1-3]. Many safety-net and public health systems serving primarily publicly insured, low-income populations [4,5], implemented ambulatory telemedicine with little prior preparation or experience [6,7]. Most safety-net sites provided mainly audio-only telephone visits, in contrast to non–safety-net sites that had prior video visit infrastructure [8]. Video visits have higher patient satisfaction than telephone consultations [9], and policy briefs [10] predict that telephone-only visits will not be reimbursed at parity with video after the pandemic abates [11,12]. As telemedicine becomes integrated into ambulatory care, safety-net health networks must explore factors affecting audio versus video telemedicine visits to maintain reimbursement parity with in-person visits.

Prior studies have found older adults, those insured by Medicaid, or low-income [13] and racial and ethnic minorities receive fewer video visits [14-16]. This has been hypothesized to be due to patient-facing barriers, such a lower digital literacy and lack of patient access to video-enabled smartphone and internet [17,18] and, to some degree, clinician-specific factors. However, a recent analysis of telephone and video visit variation found that practices (38%) and clinicians (26%) drove more of the variation in video visit use than patient-level factors (9%) [18]. System workflows are emphasized as influential for telemedicine implementation in rural sites [19]. Since much of the current literature has focused on patient-level telemedicine barriers to explain differences in telephone versus video visit uptake in the safety-net [9,18,20], we sought to assess other system-level factors influencing telephone and video visit implementation at a large urban public hospital during the first 6 months of the COVID-19 pandemic. Examination of system-level implementation factors during the early transitional phase of telemedicine can inform the adoption and sustainability of video visit use at safety-net sites [13].

## Methods

### Study Setting

Our study setting was a large, hospital-linked ambulatory clinic network serving an ethnically diverse, publicly insured patient population. This network had limited telemedicine visit capacity prior to the COVID-19 pandemic and began providing telemedicine care on March 3, 2020, with support and infrastructure primarily for telephone visits. Departments and clinicians could provide video visits on an ad hoc basis. All clinic and charting rooms had landlines and access to local area network–connected client computers without webcams. The network developed a standardized electronic health record (EHR) visit type and video visit workflow on June 15, 2020. Details of EHR implementation for telemedicine during the COVID-19 pandemic in this network are published elsewhere [21].

### Ethical Considerations

This quality improvement study was exempt from institutional review board review.

### Study Participants

We invited all 643 clinicians providing ambulatory telemedicine care to participate via email, with prompts from specialty-specific study champions. We aimed for a response rate greater than 40% given the challenges of surveying clinicians during the COVID-19 pandemic.

### Study Instrument

We conducted a cross-sectional anonymous survey via the cloud-based platform Qualtrics. We developed our survey on the basis of key constructs from validated implementation science and telemedicine surveys such as the Telehealth Usability Questionnaire [22-25] and discussions with clinical leaders in the network. The full survey is provided in Multimedia Appendix 1. We conducted pretesting of the instrument at 2 clinical sites from April 20 to May 18, 2020. We distributed the finalized survey from May 28 to July 14, 2020. Reminders were sent by department-specific champions during June 2020.

### Primary Outcome Variables: Phone and Video Visit Adoption

To assess telemedicine adoption, we asked respondents, “On average, how many telemedicine visits do you complete per half-day session? Think back to the last month of ambulatory care.” Answer options were 0, 1-3, 4-6, 7-9, or ≥10. We dichotomized responses for our two distinct outcomes of interest: (1) high telephone visit adoption (defined as 4 visits per half day or more on average in the last month) and (2) video visit adoption (defined as at least 1 video visit on average per half day in the last month at the telemedicine clinic). We defined “high telephone adopter” as 4 visits or more because telemedicine was still a novel innovation for the delivery system at this time, with almost none prior to March 2020. In June 2020, ambulatory providers had 2-12 visits total per day across specialties (mean 5 visits per day); 4 visits comprise a majority of a day’s visits and therefore fits our definition of substantial use. The half day distinction is because of the high proportion of part-time clinicians in this network. For video visits, there was extremely low uptake (<1%) of video visit use across the network [26]; therefore, even experience with 1 video visit per half day on average would signify adopters of video visits. We excluded residents and trainees as they rotated through multiple sites and had inconsistent experiences with telemedicine during this period.

### Individual-Level Clinician Characteristics

We assessed demographic characteristics including age, gender, and clinical specialty. Respondents reported their age range (<30 years, 30-49 years, etc.), which we collapsed to a binary variable of <50 versus >50. We categorized gender as male and nonmale (combining female, nonbinary or nonconforming, and transgender entries to minimize identifiability without excluding sexual or gender minorities [27]). We grouped clinical specialty as “primary/urgent care,” “medical specialty,” or “surgical specialty” (see Table 1 and Multimedia Appendix 2 for all specialty categories included in this survey).

**Table 1:** Clinician characteristics and self-reported telemedicine use (N=311).

Characteristics		Participants, n (%)
**Age (years)**		
	20-29	7 (2.25)
	30-39	47 (15.1)
	40-49	42 (13.5)
	50-59	50 (16.1)
	60-69	43 (13.8)
	≥70	52 (16.7)
	Missing or not disclosed	70 (22.5)
**Gender**		
	Female	181 (58.2)
	Male	51 (16.4)
	Nonbinary	3 (0.9)
	Transgender	0 (0)
	Missing or not disclosed	76 (24.4)
**Clinician role**		
	Faculty or attending physician	144 (46.3)
	Nurse practitioner or physician assistant	51 (16.4)
	Licensed counselor, social worker, or marriage family therapist	9 (2.9)
	Other^a^	36 (11.5)
	Missing or not disclosed^a^	71 (22.8)
**Specialty**		
	**Primary care and urgent care (total)**	**108 (34.7)**
	Family medicine	47 (15.1)
	Internal medicine	32 (10.3)
	Pediatrics	23 (7.4)
	Other primary care^a^	6 (2.2)
	**Medical specialty (total)**	**115 (37.0)**
	Psychiatry	33 (10.6)
	Obstetrics, gynecology, or midwifery	26 (8.4)
	Oncology	9 (2.9)
	Other medical specialty^a^	47 (15.1)
	**Surgical specialty (total)**	**24 (7.7)**
	Orthopedics	9 (2.9)
	General surgery and trauma	5 (1.6)
	Neurosurgery	2 (0.6)
	Other surgical specialty^a^	8 (2.6)
	Not disclosed	64 (20.6)
**Telephone visits per half day**		
	0	38 (12.2)
	1-3	96 (30.6)
	4-6	100 (32.1)
	7-9	61 (19.6)
	≥10	17 (5.5)
**Video visits per half day**		
	0	230 (73.9)
	1-3	71 (22.8)
	4-6	7 (2.3)
	7-9	0 (0.0)
	≥10	3 (0.9)
**Device used (smartphone, landline telephone, desktop, laptop, or tablet)**		
	Institutional only	77 (24.8)
	At least one personal device	163 (52.4)
	Missing	71 (22.8)

^a^See Multimedia Appendix 2 for all specialty categories included in this survey.

### System-Level Variables

System-level factors included the following: perceived workload of telemedicine compared to in-person consultations (more, same, or less), estimated time spent helping patients navigating their telemedicine visit (dichotomized to <5 or ≥5 minutes), ease of interpreter use during telemedicine (Likert scale of 1-4 points, dichotomized to more or less difficult than in-person consultations), desire for additional telemedicine training (with respect to conducting technical aspects of visits, supporting patients with low digital literacy, gathering clinical information, developing an assessment or plan, or teaching trainees), and adequacy of audio or video quality of telemedicine encounters (yes or no). We asked what devices were used for telemedicine encounters; respondents could select smartphone, landline phone, desktop, laptop, or tablet device and specify if these were personal devices or institutional (work-provided) devices; we dichotomized this to “any personal device use” versus “only institutional device use.”

### Statistical Analysis

We conducted bivariate chi-square tests to assess if individual-level characteristics and system-level variables were associated with high telephone adoption, and, separately, video visit adoption. We conducted a sensitivity analysis including only respondents who had completed ≥50% of the survey for a “complete case” analysis. There were no major differences in including all responses versus “complete case” analysis; therefore, we report all responses available.

Statistical analysis was conducted in SAS software (version 9.4). This was a voluntary study encompassing the entire source population; no power calculation was attempted given the lack of prior reference data on telemedicine during a pandemic to extrapolate to the analysis.

## Results

We collected 311 responses from 643 eligible clinicians, which resulted in a response rate of 48.4%. Full demographic characteristics of respondents are listed in Table 1. Primary and urgent care clinicians comprised 34.7%, medical specialists comprised 37.0%, and surgical specialists comprised 7.7% of the participant pool. Of clinicians, 57.2% (178/311) reported ≥4 telephone visits per half day on average. There were 81 of 311 (26.1%) clinicians who reported at least 1 video visit per half day on average. Most respondents (163, 52.4%) used ≥1 personal device (either smartphone, landline phone, laptop, desktop, or tablet device) for telemedicine encounters; 77 (24.8%) physicians used exclusively work-provided, institutional devices.

**Figure 1:**
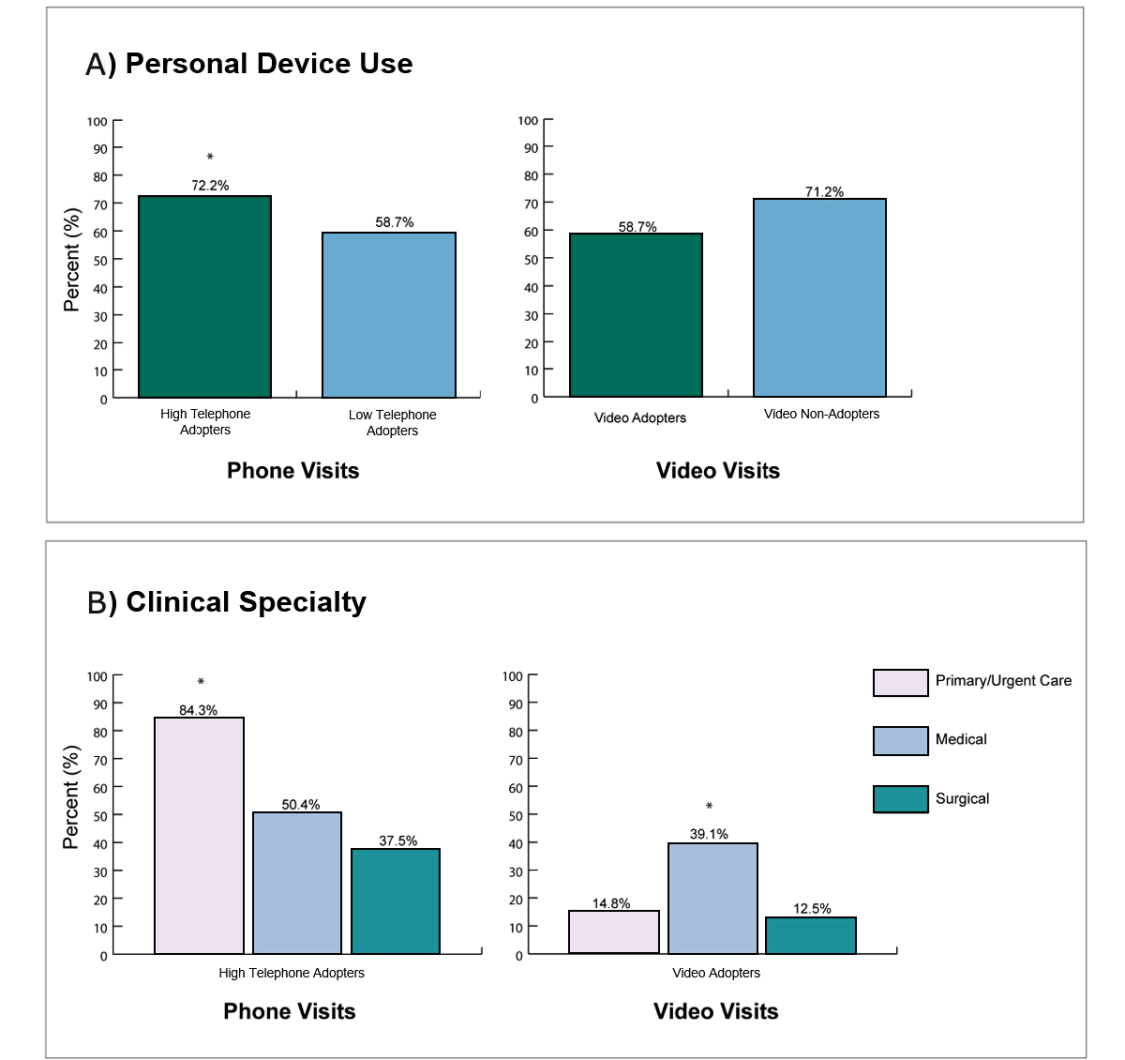
Association between clinician and system-level factors with self-reported high telephone use and any video use, comparing adopters to nonregular/nonadopters. Experience with 4 completed telephone visits in a half day session implies a high telephone adopter, and <4 implies a low telephone visit adopter. 1 video visit per half day on average signifies a video visit adopter. (A) Using at least one personal device is associated with being a high telephone adopter (72.2%, 117/162), compared to 59.0% (46/78) among low telephone adopters (*χ*^2^_1_= 4.24, *P*=.04). Personal device use was higher among video nonadopters than for video visit adopters, but this was not significant (58.7%, 37/63 vs 71.2%, 126/177; *χ*^2^_1_=3.31; *P*=.07). (B) Primary or urgent care specialty had the greatest high telephone adoption (84.3%, 91/108) compared to medical (50.4%, 58/115) and surgical (37.5%, 9/24) specialties (*χ*^2^_1_=35.7, *P*<.001). Medical specialties had the highest proportion of video adoption (39.1%, 45/115) compared to primary care (14.8%, 16/108) and surgical (12.5%, 3/24) (*χ*^2^_1_=19.64, *P*<.001).

On bivariate analysis, we found that personal device use was associated with high telephone adoption (Figure 1A; Table 2). Among high telephone adopters, 72.2% (117/163) utilized at least one personal device for telemedicine encounters (*P*=.04). Personal device use was higher among video visit nonadopters than among video visit adopters, but this was not significant (71.2%, 126/177 vs 58.7%, 37/63, *P*=.07).

**Figure 2:**
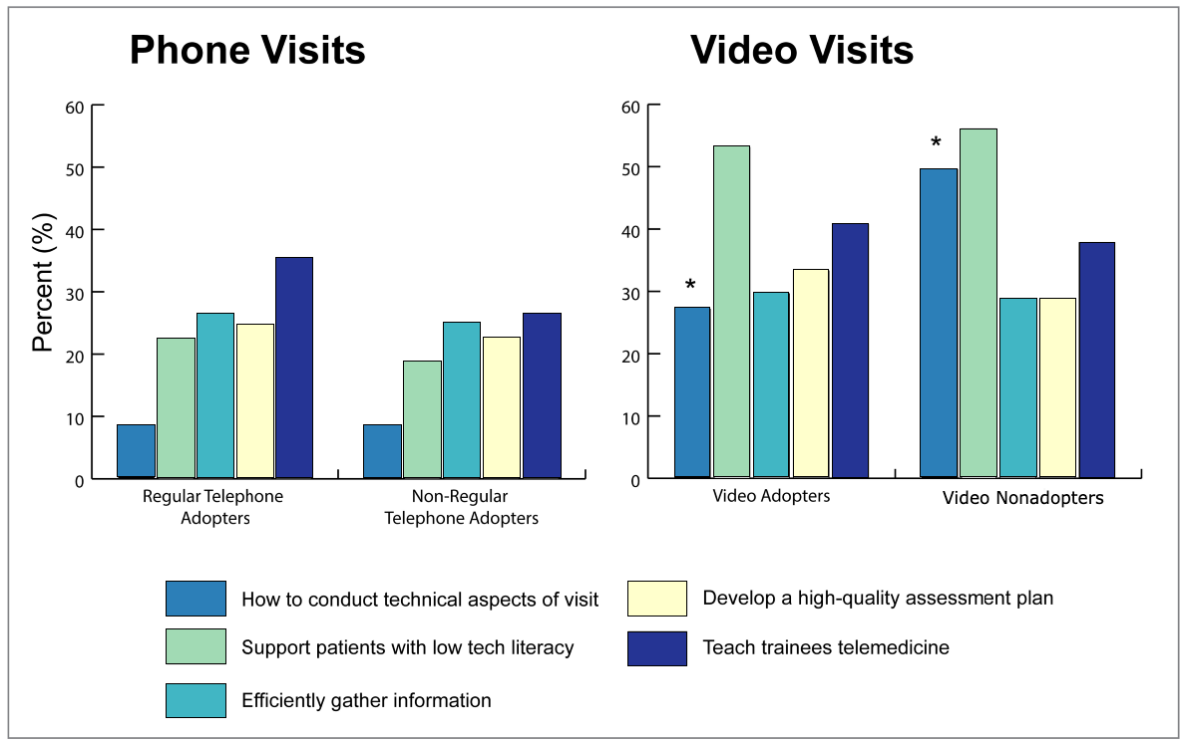
Clinician-identified training needs for conduct of telemedicine, comparing adopters to low/nonadopters. Experience with 4 completed telephone visits in a half day session implies a high telephone adopter, and <4 visits implies a low telephone adopter. Experience with 1 video visit per half day on average implies a video visit adopter. *A higher proportion of video nonadopters stated a desire for training on the technical aspects of a telemedicine visit, compared to video adopters (49.6%, 57/115 vs 27.2%, 28/103; *P*<.001).

Many video adopters as well as nonadopters expressed interest in many training domains, especially supporting patients with low technical literacy, technical aspects of the visit, and teaching trainees telemedicine (Figure 2). Desire for training in conducting technical aspects of a telemedicine visit was statistically significantly higher for video nonadopters (49.6%) than for video adopters (27.2%, *P*<.001).

We found that clinical specialty was significantly associated with high telephone adoption and video adoption (Figure 1B). Primary or urgent care clinicians had the highest proportion of high telephone adoption (84.3%, 91/108), compared to 50.4% (58/115) of medical and 37.5% (9/24) of surgical specialties (*P*<.001). Medical specialties had the highest proportion of video adoption (39.1%, 45/115), compared to 14.8% (16/108) of primary care and 12.5% (3/24) of surgical physicians (*P*<.001).

We did not find associations between telemedicine adoption and workflow-specific challenges (Table 2). The perceived workload of telemedicine relative to in-person visits, the estimated average time spent helping set up a patient for a telemedicine visit, and the perceived difficulty of working with an interpreter in a telemedicine encounter were not significantly different between telephone high-adopters or video adopters versus low or nonadopters. Few clinicians reported inadequate audio quality during telephone visits (16/166, 9.9% for high-adopters and 9/91, 12.0% for low adopters). Of video visit adopters, 8 of 70 (13.6%) found audiovisual quality inadequate.

**Table 2:** Association of system-level factors with self-reported high telephone use and any video use, the latter 2 being independent outcomes and calculated separately.

System-level factors (N=311)			Telephone visits per half day											Video visits per half day									
			≥4 visits (n=178), n/n (%)		≤3 visits (n=133), n/n (%)		Chi-square (*df*)			*P* value^a^			≥1 video visit (n=81), n/n (%)			No video visit (n=230), n/n (%)			Chi-square (*df*)			*P* value^a^	
**Perceived workload (compared to in-person visits)**								2.78 (1)			.10									1.32 (1)			.25
	Less or same workload	143/166 (86.1)		71/91 (78.0)								42/70 (60.0)			20/41 (48.8)								
	More workload	23/166 (13.9)		20/91 (22.0)								28/70 (40.0)			21/41 (51.2)								
**Time helping patients navigate (minutes)**								0.0006^a^ (1)			.98									0.51 (1)			.47
	≤4	131/154 (85.1)		69/81 (85.2)								30/62 (49.4)			13/31 (40.6)								
	≥5	23/154 (14.9)		12/81 (14.8)								32/62 (51.6)			19/31 (59.4)								
**Ease of interpreter services (compared to in-person visits)**								0.02 (1)			.90									1.16 (1)			.28
	Somewhat or much more difficult	88/134 (65.7)		34/51 (66.7)								24/31 (77.4)			17/19 (89.5)								
	Somewhat or much easier	46/134 (34.3)		17/51 (33.3)								7/31 (22.6)			2/19 (10.5)								
**Audio and video quality**								1.41 (1)			.24						N/A^b^			N/A			N/A
	Not adequate	17/161 (10.6)		12/75 (16.0)								8/59 (13.6)			N/A			N/A			N/A		
	Adequate	144/161 89.4)		63/75 (84.0)								51/59 (86.4)			N/A			N/A			N/A		
**Device**								4.24 (1)			.04^a^									3.31 (1)			.07
	Only institution-provided devices	45/162 (27.8)		32/78 (41.0)								26/63 (41.3)			51/177 (28.8)								
	At least one personal device used	117/162 (72.2)^a^		46/78 (59.0)								37/63 (58.7)			126/177 (71.2)								

^a^Significant at *P*<.05.

^b^N/A: not applicable.

## Discussion

### Principal Findings

Our investigation is one of the few studies assessing system-level factors that might influence multispecialty clinicians’ telephone and video visit adoption at a safety-net site in the early phase of the COVID-19 pandemic. Like many safety-net clinics, our telemedicine services were largely telephone-based [8].

We found that many clinicians utilize their personal devices to provide both telephone and video visits. A study of family planning clinicians found high personal smartphone use for telemedicine care during the COVID-19 pandemic, despite respondents’ preference for work-issued devices [28]. This raises equity concerns, as not all clinicians will have the same access to high-quality video and audio via their smartphone, tablet device, or computer. In our network, departments variably distributed laptops with video capacity. Privacy and security are concerns if personal devices are used for encounters without appropriate encryption, 2-factor authentication, or HIPAA (Health Insurance Portability and Accountability Act)–approved software [29]. Our system supports a server-based thin client computing environment where most computers are run from a central server. Widespread use of hardware peripherals such as webcams for video visits on such devices increases risk for network instability. This will limit safety-net clinicians who practice on site to reliably use video visits without using or compromising the security of their personal devices [30,31]. We also observed higher personal device use among video nonadopters than video adopters, although this was not significant. This may be due to clinician reluctance to access clinical or HIPAA-relevant video through their personal device, which may be an additional barrier to video visit adoption that warrants further investigation.

Desire for more training in telemedicine was high for video visits, with almost half of video nonadopters requesting training in the conduct of the technical aspects of the visit. The extent and capacity to which clinicians have been trained in telemedicine modalities have been variable throughout the COVID-19 pandemic, and standardized trainings may influence adoption [32].

Although we could not statistically compare telephone adopters against video adopters, we see that barriers such as increased workload, greater time spent supporting the patient, difficulty with the interpreter, and poor audiovisual quality were more common for video adopters than high telephone adopters. In this network, clinicians must provide their own time and support necessary to allow patients to engage in video visits. Direct patient supports, through patient orientations or external services assisting patients in logging into video visits and contacting an interpreter, would drastically reduce these barriers.

Neither perceived workload of telemedicine (compared to in-person care), nor length of time spent supporting a patient to enter a telemedicine visit were associated with telemedicine adoption. Interpreter challenges were not associated with adoption, although this has been endorsed as a barrier by clinicians [33]. Although safety-net patients may experience access barriers to bandwidth or adequate audio, audio quality was not associated with telemedicine adoption. Our sample size may have been too small to detect a relationship with these factors. Another interpretation is that clinicians using telemedicine during the COVID-19 pandemic were willing to surmount significant patient-level barriers to provide care. As telemedicine continues, it will be important to assess if later adopters will have different responses to these workload-related challenges.

Within this network, there was wide variation in infrastructural support for video visits by department. Some surgical specialties may have needed in-person evaluation rather than virtual visits. These factors likely explain specialty-specific differences in our findings.

Since our survey, telemedicine has become integrated into ambulatory care and is likely “here to stay,” both owing to ongoing pandemic surges because of new variants, as well as patient and provider preference for the convenience and access enabled by virtual visits. For example, in this network, telemedicine volume appears to be approaching a plateau of 20%-30% of primary and specialty care, although video visit uptake remains low (internal data). Learning health systems serving vulnerable populations must assess the first years of implementation to optimize video visit delivery for the long term. Without system-level investments, disparities in video visit access may worsen disparities in the safety-net [34]. First, safety-net leaders should assess telemedicine utilization patterns by department to perceive variation in audio-only versus video visits. Reliance on personal device use should be surveyed; future research should query what devices are key for high-quality telemedicine interactions [35,36]. Third, further research should explore facilitators and barriers to video visits in safety-net settings, and identify what skills and trainings are most helpful to support telemedicine practice. Clinician ability to provide video visits will remain a priority in a shifting reimbursement policy landscape.

### Limitations

We almost reached a 50% response rate for this voluntary survey among diverse clinicians. Although this was greater than our prespecified target, participants may have differed in their experience of telemedicine compared to nonparticipants. There was high variation in the completeness of survey responses; this was likely owing to the survey burden early during the pandemic. However, we observed no differences in results when comparing all participants to complete case analysis. Owing to survey missingness, we could not generate a multivariable model nor assess for variable interactions. As findings are based on bivariate analysis, they are exploratory and hypothesis-generating for system-level factors that may be related to safety-net telemedicine implementation. As telemedicine workflows were evolving during the survey period, some telemedicine practices may have changed during our assessment; this may have confounded some of our findings regarding specialty-specific differences. However, although a specific workflow was disseminated in June 2020, the same video visit software was available to clinicians over the entire survey period. While our definitions of “high telephone adoption” and “video adoption” were appropriate when we conducted the survey, telemedicine volume will continue to shift and evolve. Comparing the proportion of telemedicine visits to that of in-person visits may be an alternative definition for future studies; qualitative work should also assess how varying specialties choose to optimize their ambulatory schedules between virtual and in-person visits.

### Conclusions

Clinical specialty type, personal device use, and desire for technical training were major factors associated with telephone and video visit adoption among safety-net clinicians. Department-level support, assessment of use of personal devices, and clinician training are priorities for safety-net systems.

## Appendix

Multimedia Appendix 1 Survey instrument.

Multimedia Appendix 2 Respondent reported clinical specialities.

## Abbreviations

EHR: electronic health record
HIPAA: Health Insurance Portability and Accountability Act
