# Supplementary material for: System-Level Factors Associated With Telephone and Video Visit Use: Survey of Safety-Net Clinicians During the Early Phase of the COVID-19 Pandemic
Source: JMIR Form Res. 2022 Mar 10;6(3):e34088. doi: 10.2196/34088 (PMC8949684; doi:10.2196/34088)
Supplement: Multimedia Appendix 1 [file formative_v6i3e34088_app1.docx]

Supplementary File 1: Survey Instrument

UCSF ZSFG Telemedicine Implementation Survey


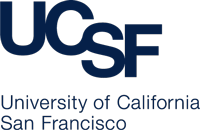


**Telemedicine utilization/experience thus far**

Due to the COVID-19 pandemic, we rapidly expanded telemedicine care. We are conducting a short, anonymous survey for quality improvement. Your responses will help us improve our telemedicine services for providers across ZSFG and DPH.

Please complete if you deliver ambulatory care for ZSFG and DPH patients. This survey should take no more than 7 minutes. For any questions, contact anjana.sharma@ucsf.edu or delphine.tuot@ucsf.edu. Thank you!

On average, how many telemedicine visits do you complete per ½ day session? Think back to the last month of ambulatory care.

|  | 0 (1) | 1-3 (2) | 4-6 (3) | 7-9 (4) | 10+ (5) |
| --- | --- | --- | --- | --- | --- |
| Telephone (1) |  |  |  |  |  |
| Video (2) |  |  |  |  |  |

**Workﬂow/Usefulness**

How does telemedicine affect OVERALL provider workload, compared to a traditional in-person visit? This includes chart prep, documentation, communication with staff, and follow-up. Answer based on your experience or how you think it has affected colleagues in a similar position to you.

|  | More workload overall (1) | Workload is the same overall (2) | Less workload overall (3) | N/A (4) |
| --- | --- | --- | --- | --- |
| Telephone (1) |  |  |  |  |
| Video (2) |  |  |  |  |

How useful are telemedicine visits for the following types of encounters? Complete based on your experience or your impressions.

|  | Telephone | | |  |  |  |
| --- | --- | --- | --- | --- | --- | --- |
|  | Very useful (1) | Somewhat useful (2) | Not very useful (3) | | Not useful at all (4) | N/A (5) |
| Generating a new diagnosis or treatment plan (1) |  |  |  | |  |  |
| Discussing test results with patients (2) |  |  |  | |  |  |
| Management of known diagnosis (3) |  |  |  | |  |  |

|  | Video | | |  |  |  |
| --- | --- | --- | --- | --- | --- | --- |
|  | Very useful (1) | Somewhat useful (2) | Not very useful (3) | | Not useful at all (4) | N/A (5) |
| Generating a new diagnosis or treatment plan (1) |  |  |  | |  |  |
| Discussing test results with patients (2) |  |  |  | |  |  |
| Management of known diagnosis (3) |  |  |  | |  |  |

**Safety Implications**

Please tell us whether you agree or disagree: **Compared with in-person visits,** I’m concerned about the **safety** of telemedicine because of increased risk of missed or delayed diagnosis during...

|  | Strongly agree (1) | Agree (2) | Disagree (3) | Strongly disagree (4) |
| --- | --- | --- | --- | --- |
| Telephone visits (1) |  |  |  |  |
| Video visits (2) |  |  |  |  |

**Provider Satisfaction**

*Shown to respondents who had any telehealth (video or phone).*

Please rate your **satisfaction with your clinical care** during telemedicine sessions compared with in-person visits.

|  | Extremely satisfied (1) | Somewhat satisfied (2) | Somewhat dissatisfied (3) | Extremely dissatisfied (4) |
| --- | --- | --- | --- | --- |
| Telephone (1) |  |  |  |  |
| Video (2) |  |  |  |  |

Please rate your **satisfaction with your clinical care** during telephone sessions compared with in-person visits.

- Extremely satisfied (1)
- Somewhat satisfied (2)
- Somewhat dissatisfied (3)
- Extremely dissatisfied (4)

Please share an example of something that **went well** during a telemedicine visit:

- Telephone (1) ________________________________________________
- Video (2) ________________________________________________

Please share an example of something that **went well** during a telephone visit:

________________________________________________________________

________________________________________________________________

________________________________________________________________

________________________________________________________________

________________________________________________________________

Please share an example of something that **did not go well** during a telemedicine visit:

________________________________________________________________

________________________________________________________________

________________________________________________________________

________________________________________________________________

________________________________________________________________

**Patient Access**

How much time per encounter do you have to help your patients navigate the phone or video technology for these sessions?

|  | 0-1 minutes (1) | 2-4 minutes (2) | 5-10 minutes (3) | 10+ minutes (4) | N/A (5) |
| --- | --- | --- | --- | --- | --- |
| Telephone visits (1) |  |  |  |  |  |
| Video visits (2) |  |  |  |  |  |

Please select any **patient challenges to accessing telemedicine care** that you have observed:

|  | Telephone visit (1) | Video visit (2) |
| --- | --- | --- |
| Lack of knowledge or skills to participate in the visit (1) |  |  |
| Lack of phone (2) |  |  |
| Lack of internet (3) |  |  |
| Lack of video (4) |  |  |
| Trouble using apps (5) |  |  |
| Speech, hearing, or cognitive barrier (6) |  |  |
| Diminished patient comfort or trust (7) |  |  |
| Communication quality (8) |  |  |
| Scheduling difficulties (9) |  |  |
| Lack of privacy or confidentiality (10) |  |  |
| Other (11) |  |  |

**Educational needs/interest**

Please describe your experience working with interpreter services during telemedicine visits, compared to in-person visits.

|  | Much more difficult (1) | Somewhat more difficult (2) | Somewhat easier (3) | Much easier (4) | N/A (5) |
| --- | --- | --- | --- | --- | --- |
| Telephone (1) |  |  |  |  |  |
| Video (2) |  |  |  |  |  |

Please **describe your comfort level** with providing telemedicine care using...

|  | Very comfortable (1) | Somewhat comfortable (2) | Somewhat uncomfortable (3) | Extremely uncomfortable (4) |
| --- | --- | --- | --- | --- |
| Telephone (1) |  |  |  |  |
| Video (2) |  |  |  |  |

I desire additional **training or education in how to**:

|  | Telephone (1) | Video (2) |
| --- | --- | --- |
| Conduct technical aspects of telemedicine (connect to patient, run software) (1) |  |  |
| Support patients with low technological literacy (2) |  |  |
| Efficiently gather clinical information during telemedicine visit (3) |  |  |
| Develop a high-quality assessment and plan for telemedicine visit (4) |  |  |
| Teach trainees while using telemedicine with patients (5) |  |  |
| Other (6) |  |  |

**Intention for future use/feasibility**

Please select any equipment you currently use for telemedicine care.

|  | Who provided this equipment? | | |
| --- | --- | --- | --- |
|  | Personal (1) | DPH (2) | UCSF (3) |
| Office (landline) (1) |  |  |  |
| Smartphone (2) |  |  |  |
| Desktop (3) |  |  |  |
| Laptop (4) |  |  |  |
| Tablet (5) |  |  |  |

Is the **audio or video quality** you've experienced adequate for telemedicine patient care?

|  | Yes (1) | No (2) | N/A (3) |
| --- | --- | --- | --- |
| Telephone (1) |  |  |  |
| Video (2) |  |  |  |

Is the **audio quality** you've experienced adequate for telemedicine patient care?

- Yes (1)
- No (2)
- N/A (3)

When clinical operations return to normal, how likely are you to continue performing some telemedicine visits by choice?

|  | Very likely (1) | Somewhat likely (2) | Not at all likely (3) |
| --- | --- | --- | --- |
| Telephone (1) |  |  |  |
| Video (2) |  |  |  |

Please share any other perspectives or comments about your experiences with telemedicine that haven't been covered by this survey. (Optional)A

________________________________________________________________

________________________________________________________________

________________________________________________________________

________________________________________________________________

**Demographics**

What is your **primary** **clinic site/medical specialty**?

- Adult Urgent Care (1) 1
- Anticoagulation Clinic (2) 3
- Breast Surgery (3) 0
- Burn/Wound Clinic (4)
- Cardiology (5)
- Cardiothoracic Surgery (6)
- Colorectal Surgery (7)
- Dermatology (8)
- Diabetes Clinic (9)
- Endocrinology (10)
- Family Medicine (11)
- Gastroenterology (12)
- General Surgery & Trauma (13)
- Geriatrics (14)
- Hematology (15)
- Hepatology (16)
- Infectious Diseases (17)
- Internal Medicine Primary Care (18)
- Lipid Clinic (19)
- Nephrology (20)
- Neurology (21)
- Neurosurgery (22)
- Ob/GYN/Midwifery (23)
- Oncology (24)
- Oral Surgery (25)
- Orthopedics (26)
- Ophthalmology (27)
- Optometry (28)
- Otolaryngology (29)
- Pain Clinic (30)
- Palliative Care (31)
- Pediatrics (32)
- Pediatric Asthma/Allergy (33)
- Pediatric Cardiology (34)
- Pediatric Dermatology (35)
- Pediatric Healthy Lifestyles (36)
- Pediatric Neurology (37)
- Pediatric Nutrition (38)
- Pediatric Urgent Care (39)
- Pediatric Urology (40)
- Oncology (41)
- Plastic Surgery (42)
- Podiatry (43)
- Psychiatry (44)
- Pulmonology (45)
- Rheumatology (46)
- Urology (47)
- Vascular Surgery (48)
- Weight Management (49)
- Other/Not Listed (50)

What is your **gender**? (You can select all that apply)

- Female (1)
- Male (2)
- Non-binary or gender non-conforming (3)
- Transgender (4)
- Prefer to self-describe: (5)
- Prefer not to say (6)

What is your **age range**?

- 20-29 (1)
- 30-39 (2)
- 40-49 (3)
- 50-59 (4)
- 60-69 (5)
- 70 or older (6)

How many years have you been in practice (years since completing training/residency/fellowship)?

- Currently in training (1)
- 1-5 years (2)
- 6-10 years (3)
- 11-15 years (4)
- 16-20 years (5)
- 21+ years (6)

What is your **role**?

What is your role?

- NP or PA (1)
- Pharmacist (2)
- Resident (3)
- Fellow (4)
- Faculty/attending (5)
- Nurse Midwife/CNM (6)
- CRNA (7)
- DDS (8)
- DPM (9)
- Licensed Counselor (10)
- OD (11)
- PsyD (12)
- Other (13) ________________________________________________

Thank you for your responses and for your care of our patients.
