# Supplementary material for: System-Level Factors Associated With Telephone and Video Visit Use: Survey of Safety-Net Clinicians During the Early Phase of the COVID-19 Pandemic
Source: JMIR Form Res. 2022 Mar 10;6(3):e34088. doi: 10.2196/34088 (PMC8949684; doi:10.2196/34088)
Supplement: Multimedia Appendix 2 [file formative_v6i3e34088_app2.docx]

**Multimedia Appendix 2.**

**Respondent Reported Clinical Role and Specialties (n=311)**

| SPECIALTY |  |
| --- | --- |
| Primary Care/Urgent | 108 (34.7) |
| Adult Urgent Care | 1 (0.3) |
| Anticoagulation Clinic | 3 (1) |
| Family Medicine | 47 (15.1) |
| Geriatrics | 1 (0.3) |
| Internal Medicine | 32 (10.3) |
| Pediatric Urgent Care | 1 (0.3) |
| Pediatrics | 23 (7.4) |
| Medical Specialty | 115 (37) |
| Cardiology | 6 (1.9) |
| Dermatology | 3 (1) |
| Diabetes Clinic | 4 (1.3) |
| Endocrinology | 2 (0.6) |
| Gastroenterology | 3 (1) |
| Hepatology | 2 (0.6) |
| Infectious Disease | 6 (1.9) |
| Nephrology | 3 (1) |
| Neurology | 1 (0.3) |
| OB/GYN/Midwifery | 26 (8.4) |
| Oncology | 9 (2.9) |
| Pain Clinic | 2 (0.6) |
| Palliative Care | 5 (1.6) |
| Pediatric Asthma/Allergy | 1 (0.3) |
| Pediatric Neurology | 1 (0.3) |
| Psychiatry | 33 (10.6) |
| Pulmonology | 4 (1.3) |
| Rheumatology | 4 (1.3) |
| Surgical Specialty | 24 (7.7) |
| General Surgery & Trauma | 5 (1.6) |
| Neurosurgery | 2 (0.6) |
| Orthopedics | 9 (2.9) |
| Ophthalmology | 1 (0.3) |
| Optometry | 2 (0.6) |
| Pediatric Urology | 1 (0.3) |
| Podiatry | 2 (0.6) |
| Urology | 1 (0.3) |
| Vascular Surgery | 1 (0.3) |
| Specialty Not Disclosed | 64 (20.6) |
| Total | 311 |
| CLINICIAN TYPE |  |
| Faculty/attending physician | 144 (46.3) |
| Nurse practitioner/Physician Assistant | 51 (16.4) |
| Licensed counselor/Social worker/marriage family therapist | 9 (2.9) |
| Psychologist | 9 (2.9) |
| Nurse midwife | 7 (2.3) |
| Pharmacist | 6 (1.9) |
| Non-ACGME Fellow | 5 (1.6) |
| Occupational therapist/Speech language pathologist | 3 (1.0) |
| Genetic counselor | 2 (0.6) |
| Optometrist | 2 (0.6) |
| Acupuncturist | 1 (0.3) |
| Other | 1 (0.3) |
| Missing/Not disclosed | 71 (22.8) |
